# Pediatric allergy and immunology for patients and parents: challenges of developing website and social network during COVID-19 pandemic in Brazil

**DOI:** 10.1590/1984-0462/2023/41/2022032

**Published:** 2023-03-13

**Authors:** Marina Mayumi Vendrame Takao, Fabiana Silveira de Souza, Luísa Riccetto, Rosana Evangelista-Poderoso, Adriana Gut Lopes Riccetto, Marcos Tadeu Nolasco da Silva

**Affiliations:** a Universidade Estadual de Campinas, Campinas, SP, Brazil.; b Pontifícia Universidade Católica de Campinas, Campinas, SP, Brazil.

**Keywords:** Health education, Telemedicine, Internet, Allergy and immunology, Educação em saúde, Telemedicina, Internet, Alergologia e imunologia

## Abstract

**Objective:**

To describe the development of a website and the creation of a social network account about pediatric allergy/immunology with reliable information, to promote education and have a channel for patient-doctor contact.

**Methods:**

This is a descriptive study. A survey was conducted with 93 patients (12 years and older) and caregivers of a Pediatric Allergy/Immunology outpatient clinic, to assess internet usage patterns of potential users. A webpage in Portuguese and an Instagram^®^ account were launched in which it was created an area for patient-doctor communication in the pandemic context.

**Results:**

Among 93 participants, 77% were female, 82% caregivers. Median age was 33.2 years, family income 403 dollars/month. The internet was accessed via smartphone by 81,7% of the participants; 76% reported using internet to access health information but 72% did not trust on the information from the internet, and 96% believed that an institutional site could provide meaningful information. From the website release in November 6, 2018 to January 20, 2022, it was counted 10,062 page views by 4,896 users; 55% were 18–34 years old, 70.2% female. Instagram^®^ account gathered 882 followers. Website went through a period of instability during which access were not counted. Due to social isolation during COVID-19 pandemic, the website served as a tool for first response to help patients and doctors.

**Conclusions:**

Patients and caregivers of the Pediatric Allergy/Immunology service, consulted about digital tools, considered the information supported by a teaching/research institution timely and relevant. The website and Instagram^®^ account have both performed well and shown good return in relation to hits, and results are continuously being evaluated. During COVID-19 pandemic, the website has been connecting patients/families and doctors.

## RESUMO

**Objetivo:**

Descrever o desenvolvimento de um *website* e a criação de uma conta em rede social sobre alergologia/imunologia pediátrica com informações confiáveis, para promover educação e ter um canal de contato paciente-médico.

**Métodos:**

Trata-se de um estudo descritivo. Um questionário foi aplicado a 93 pacientes (12 anos de idade ou mais) e cuidadores frequentadores de um ambulatório de alergologia/imunologia pediátrica, para abordar o padrão de uso de internet de potenciais usuários. Lançou-se um *website* em português e uma página no Instagram^®^, nos quais foi criada, no contexto da pandemia, uma área para comunicação paciente-médico.

**Resultados:**

Dos 93 participantes, 77% eram mulheres, 82% cuidadores, com idade mediana de 33,2 anos e renda mensal de 403 dólares. A internet foi acessada via *smartphone* por 81,7% dos participantes; 76% relataram usar a internet para informações em saúde, mas 72% não confiavam nessa informação, e 96% acreditavam que um *site* institucional poderia prover informações seguras. Do lançamento do *website* em 6 de novembro de 2018 até 20 de janeiro de 2022, contabilizaram-se 10.062 visualizações de páginas por 4.896 usuários; 55% tinham idade entre 18 e 34 anos, 70,2% eram mulheres. A página no Instagram^®^ reuniu 882 seguidores. O *website* apresentou período de instabilidade, sem registrar acessos. Em razão do isolamento social durante a pandemia de COVID-19, o *website* apresentou-se como ferramenta de primeira linha para auxiliar pacientes e médicos.

**Conclusões:**

Pacientes e cuidadores do ambulatório de Alergologia/Imunologia Pediátrica, consultados sobre ferramentas digitais, consideraram as informações fornecidas por uma instituição de ensino/pesquisa como oportunas e relevantes. O *website* e a página do Instagram^®^ têm apresentado bom desempenho e visualização, e os resultados estão sendo continuamente avaliados. Durante a pandemia de COVID-19, o *website* tem conectado pacientes/familiares e médicos.

## INTRODUCTION

In the latter years, we have witnessed a remarkable expansion of internet; smartphones and mobile health applications have become part of daily life. The way people acquire health information and how they are presented have changed — they were dominated by healthcare professionals and transmitted in formal consultations, and became public, permanent, and easily accessible. As a consequence of the COVID-19 pandemic, this reality was reinforced; accessing online health information became a need in the context of social isolation, that prevented families and patients to attend to presential medical consultations.

This reality presents positive aspects, as the ability to access, understand, evaluate, and communicate information is critical to enhance the control of individuals over their health.^1^ Patients/caregivers who use online tools feel better prepared for consultations, rise more relevant questions, know more about their healthcare and are more likely to take steps to improve their health.^2^

However, seeking health information online is not always beneficial. A systematic review about the quality of health websites concluded that 70% have problems on the quality of information.^3^ In the era of social medias and pandemic, the spreading of misinformation have become so broad and uncontrolled, that was named “Infodemics” (i.e., misinformation epidemic/pandemic) by the Director-General of the World Health Organization.^4^ The speed and absence of standards with which information is disclosed on the internet make impracticable the peer-review process, a hallmark of high-quality scientific journals.^5^

Frequently, online health information is inaccurate, incomplete, tendentious, outdated, hard to access, constituting a potential risk to the health of individuals.^6,7,8,9^ A review demonstrated that the potential harm caused by inaccurate online information encompasses physical, emotional, and financial damages.^10^ Users are not always able to distinguish which websites have credibility.

Considering the reality exposed, digital tools for spreading information developed by a specialized medical team, according to the needs identified among patients, with materials based on scientific literature and friendly presentation, represent useful tools to enhance the quality of health education and assistance. This study presents the development of a website and Instagram^®^ account with information about Pediatric Allergy and Immunology (PAI) directed to patients and families, and the way these digital tools help facilitate the connection between patients, families, and doctors during social isolation.

## METHOD

The development of the website was performed in five stages, after the approval from the Research Ethics Committee (Statement #2.718.974, June 18^th^, 2018).

Stage 1: A survey form was developed and applied to evaluate demographic and internet usage profile, as well as expectations about a website containing PAI information. A convenience sample of 93 participants was defined. Inclusion criteria comprised patients aged 12 years or more or their caregivers, on follow-up in the PAI outpatient clinic. Exclusion criteria comprised patients aged less than 12 years, patients/caregivers incapable of understanding the survey form, and those who refused to participate in the study. The survey forms were self-applied right after the medical consultations, during two months.

Stage 2: Data from the survey were tabulated (software Excel^®^) and statistical analysis was performed using descriptive measures of dispersion (software Statistical Package for the Social Sciences — SPSS^®^).

**Figure 1.**
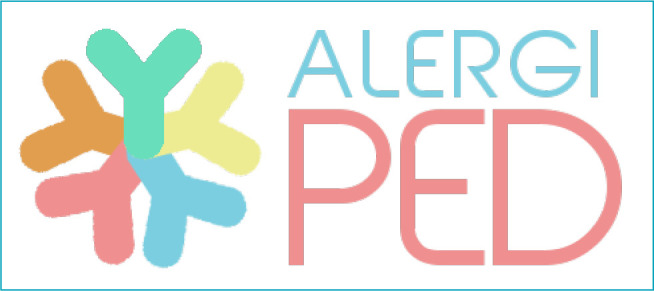
AlergiPed^®^ trademark.

Stage 3: A friendly site was developed, as our focus is on PAI (Figure 1) and on the website structure, in association with the Informatics Department of our institution.

The first version of AlergiPed^®^ website was launched on November 6th, 2018. We used the system Drupal 8^®^ for the content management of the website and MySQL^®^ for the digital data bank management. The theme “Allergic rhinitis” was the prototype chosen. Progressively, contents for the webpage were produced and published, comprising easily readable texts about allergies and immunodeficiencies (based on scientific evidence), educative videos, a list of trustworthy scientific websites for families, and a public channel for information on demand managed by the medical team. Figures were acquired in open photos platforms or through research on Google^®^, with license filters; credits were given.

Regarding the channel for questions, a statement elucidated that the answers would be provided in up to seven days and would not solve urgencies. An institutional e-mail was created to receive the demands and the users received the answers by their own e-mail. The Google Analytics^®^ was installed on the website to collect data about the access; it is one of the most sophisticated Web analytic tool that can provide quantitative and qualitative data about the users of the website, which are used for enhancing the performance of the website.^11,12,13,14^

Finally, the website was converted to smartphone-friendly interface. The page underwent modifications as it presented instabilities/errors, considered typical in the informatics area.

Stage 4: To disseminate the webpage, we chose different paths: social networks (Whatsapp^®^ and Instagram^®^), verbal form during social meetings and academic classes, and adhesives and cards distributed in the outpatient clinic. The Instagram^®^ account complements the website, and posts are produced with the application Canvas^®^, using a free account, with a short text and a photo from the application or personal collection. This process of disseminating the website will continue as long as the website is on air.

Stage 5: The impact of the website was evaluated using Google Analytics^®^. Although this tool was launched to be used in marketing/commercial areas, several health studies have been using it for web analytics, once it collects information anonymously and presents them as aggregated data, which makes it accessible in the research scenarios without ethical concerns.^12^ Finally, a request for registering the trademark AlergiPed^®^ was submitted to the institutional Agency of Innovation.

## RESULTS

The survey was answered by 93 participants, most of them were female (77%) and mothers of the patients (72%). Their median age was 33.2 years, median schooling was 11 years (complete high school), and median monthly income was 403 US dollars (which represents 146.5 dollars above the minimum wage in Brazil at the time). Regarding the use of internet, 97% reported having access, two third use smartphones. The median frequency of access was seven days/week, three hours/day (Table 1). As for the research of health topics on the internet, 76% affirmed to have done it already. The same percentage preferred to use research sites like Google^®^. In relation to the use of information obtained online, 18% of the users stated they always trusted them, 53% believed they knew how to distinguish reliable information from false ones, and 37% had already practiced health information acquired online. Finally, almost all the participants (97%) believed AlergiPed^®^ would help to understand the health condition and care with children, and 90% would access it, hoping to find information about the cause and treatment of immunologic diseases, presented through texts, images, and videos.

**Table 1.** Social, demographic and use of internet characteristics of interviewed population.

	Valid (n=93)	Missing	Median	Minimum–maximum
Age (years)	88	5	33.2	11.8–72.1
Schooling (years)	84	9	11	2–17
Monthly income (U.S. Dollars)	81	12	403	133–1,905
Days of access/week	86	7	7	2–7
Hours of access/day	79	14	3	1–19

A quantitative analysis of the accesses to the website was done with Google Analytics^®^ since the website release on November 6^th^, 2018, including the COVID-19 pandemic period. From March 2021 to November 2021, the analysis presented instabilities due to errors on the communication of our website with Google Analytics^®^, consequently, accesses and users were not fully registered. During this period, the website was not off-air. Informatics team managed to correct these instabilities, but the data lost in this gap could not be recovered.

From the website release until January 20^th^, 2022, it was identified 4,896 new users, 5,643 sessions (period in which there is active user interaction with the page) and 10,062 page views. At each session, the medium number of pages visited was 1.78 and the medium duration of each session was two minutes and 53 seconds. Regarding the demographic profile of visitors, 55% were aged between 18 and 34 years, with remarkable difference between the sexes (70.2% female and 29.8% male). As to the geographic location of users, 93.4% were in Brazil, the others were in nine other countries. Most of the users accessed the website using smartphones (81.7%). Mobile internet network was largely used. Except for the homepage, the most visited pages, in decrescent order, were: presentation of allergies, presentation of other immunologic diseases, and presentation of the team.

For a comparative analysis, from the correction of instabilities on November 15^th^, 2021 to January 20^th^, 2022 (therefore, totalizing 66 days), it was registered 4,520 new users, 4,949 sessions and 6,527 page views. Demographic and geographic profiles were very alike, as well as the device used for access: 54.9% of users were aged between 18 and 34 years, 70.2% were female, 93.1% were located in Brazil, and 81.6% used smartphone to access the website.

There was an expansion in the social media. The Instagram^®^ account was initially produced to release the website. But, due to the available tools and the agile character of social media, the initial objective was extrapolated, and nowadays, the social media is also used to update health information and interact with users, following the fundamentals of the AlergiPed^®^ project, that is to provide quality knowledge for the population. Our page has 875 followers, and we work to keep posting new contents regularly.

The registration request for AlergiPed^®^ was sent on November 11^th^, 2019. On December 29^th^, 2020 it was granted by the Brazilian National Institute of Industrial Property.

Regarding the transformations and adaptions due to COVID-19 pandemic, the website was produced to spread information about allergy and immunology, at first. In view of the social isolation brought about by COVID-19 pandemic, our outpatient clinic was suddenly closed for undetermined time. Patients had their consultations canceled, including those with severe cases of allergies and immunodeficiencies. For this reason, our team redirected the website to help spread reliable information on COVID-19 news and information about the outpatient clinic operation. Our institutional e-mail, previously attached to the channel for questions on demand, became the official means of communication with our patients who needed specialized medical guidance, and other doctors, since we could not receive them in our outpatient clinic. Even with the gradual return of presential activities, the e-mail is our main channel of communication with our patients. This effort was part of an institutional initiative of remote guidance for about 4,000 pediatric patients from different specialties who had their medical appointments canceled due to the COVID-19 pandemic.

## DISCUSSION

The survey performed in our study demonstrated a population mostly composed by mothers, adolescents/young adults who accessed the internet daily using smartphones, accessed online heath information, and considered the creation of a website with graphic features positive. After the digital initiatives were released, these media increasingly received more accesses, presented operational problems, as well as transformations imposed by the COVID-19 pandemic.

The profile of the population who responded to the survey provided important insights to structure the website. The conduction of a survey to study the preference of users is suggested by Walker et al. in a recent editorial^15^ as a tool to customize patient education, considering that a huge range of different educations would be possible, from detail to big picture, from word based to visual. The complexity of language, visual appeal, and presentation of the contents (texts, videos, photos) were guided by the demographic profile and preferences of the participants. Furthermore, according to Brazilian reality, most of the population has limited access to internet and uses smartphones for this, besides, since 2018, the exclusive use of smartphones for internet access has surpassed the combined use of computer and smartphones.^16^ The device can help manage public and individual health: applications and websites accessed by smartphones can improve the care for chronic diseases, the interaction between patients and the medical team, and the access to distant health services.^17^ Therefore, we assured that our website was adapted to smartphones and its contents did not demand excessive data. It is worth remembering that this project was carried out in one of the most developed regions of Brazil, which means that its impact may be different in economically and educationally disadvantaged communities.

The reliability of the AlergiPed^®^ website, due to scientific basis and affiliation to an academic institution, was a crucial factor for the efficacy of the access, with quality and equity. It was also our team's goal to simplify the presentation of information, with the development of a friendly interface, preserving credibility and quality of the content, once websites or applications rich in reliable information, but without a friendly browsing, cause negative impact on the perception of efficacy by users, affecting the willing to use the resource.^18^

In the present survey, 82% of the participants were caregivers, especially mothers. It reflects a Brazilian reality, as according to the 2019 National Household Sample Survey (PNAD)^19^ and the 2011 National Policy for Integral Attention to Women's Health Care (PNAISM),^20^ women correspond to most of the Brazilian population (51.8%) and to the main users of our Unified Health System (SUS). They attend healthcare facilities for their own consultations, but most importantly, they accompany children and other family members. Caregivers can benefit from internet-based resources, even though there are still gaps in the knowledge of their needs. Studies on the use of digital tools by caregivers of pediatric patients are scarce in the medical literature. Park et al.^21^ gathered 17 studies about the use of online resources by caregivers of children and adolescents, of which two were directed to asthma. In this group, most of the caregivers were also mothers. The methodological quality was considerably variable as the internet access rate, which ranged from 11 to 90%. Information about specific diseases helped to take treatment decisions, whereas social support in virtual communities helped to understand the emotional needs of caregivers. The frequent findings of those studies were the research in generic websites and the insecurity related to the capacity to evaluate the quality of information. A significant percentage of caregivers used the acquired information to dialogue with healthcare providers. Therefore, authors concluded that caregivers need better orientation on how to access health information online, and that this information should be presented in low complexity.

Similarly, a qualitative study evaluated the perspectives of online research on health information by caregivers.^22^ Aspects raised in the interviews were the difficulties and concerns in choosing reliable websites, the possibility of empowerment given by the information acquired online, clarity, and user-friendly interface of websites. Results from both studies previously cited confirm the importance of the characteristics chosen to be part of AlergiPed^®^, such as institutional reliability, interface simplicity, and content exposure.

In our study, 18% of interviewed subjects were adolescents or young adults. A systematic review^23^ identified that more than 50% of them look for online health information, being internet the most frequent source. The population of the review valued the impression of seriousness and frequent updating of the website, and they considered essential the credibility and privacy. Less instructed teenagers had more difficulty distinguishing reliable information from non-reliable. The elements from this systematic review^23^ were also considered to improve AlergiPed^®^.

Furthermore, beyond its initial scope, AlergiPed^®^ served as a tool for a first response to the chaos and limitation brought about by the sudden social isolation during the COVID-19 pandemic — a time when digital interactions replaced many face-to-face meetings and caused people across the globe to embrace digital technology.^15^ The associated Instagram^®^ account is also growing, which is expected, as the use of social media platforms increased 20–87% worldwide during the pandemic crisis.^24^

The main limitations of this initiative include the fact that the survey form was applied to a convenience sample without validation and could not be expanded due to limited availability from both patients/caregivers and researchers. In addition, the evaluation of the impact until now was based on use/access of the website, not using other tools, as during COVID-19 pandemic the communication with patients/caregivers was greatly reduced. Presently, as the routine of face-to-face consultations is completely reestablished in our service, small group meetings with caregivers and patients could help meet the specific needs of the target population, and evaluate the impact of the initiative. Technical aspects also limited the study. Ever since our website and social network were launched, our team has been continuously working to keep them in operation by publishing new contents, making regular system updates, and correcting eventual bugs. The communication failure between Google Analytics^®^ and the website, from March to November 2021, hindered us from fully collecting information about accesses and users in this period, but data from the last 66 days before the submission of this paper showed a representative increase of users and accesses, which indicates that the website has been enhancing its capacity to reach out to our public. It is important to highlight that this bug in registration was just one of various errors that happened in the period, which are expected in the informatics context and that demonstrate the challenges of maintaining a website.

The development of the AlergiPed^®^ was an opportunity to experience a significant interdisciplinary collaboration. Some of the necessary skills involved medical knowledge, communication, graphic design, programming language, network technology, data management. Considering the previous know-how of the team, it demanded effort to adapt to the new performance scenario. Our experience is consistent with a systematic review^25^ on interdisciplinary collaboration in health informatics with studies published during 25 years. Even though the focus of the researchers was not the development of online health education, authors concluded that the union of different knowledge is essential for the informatics community to provide qualified healthcare, and there is still a lack of process standardization on how this collaboration can be orchestrated.

It is possible to observe in literature that the project AlergiPed^®^ presents similarities with other projects of health education from academic institutions, but to our knowledge, this is the first website about PAI directed for families produced by Brazilian doctors.

In conclusion, the study presented the development process of a website and Instagram^®^ account AlergiPed^®^, including the stages of production, the team's difficulties and expectations related to this process, and also, the transformations imposed by social isolation during the COVID-19 pandemic. The population interviewed considered relevant the availability of these digital tools with reliable information, friendly interface, and the support of an academic institution. Results suggest that the construction and dissemination of the website and social media is a beneficial, continuous, changeable, and challenging process.
